# A Fracture Case

**Published:** 1880-03

**Authors:** F. Peterson


### A FRACTURE CASE

SERVICE OF DR. C. C. F. GAY.—REPORTED BY DR. F. PETERSON.

*Editors Buffalo Medical and Surgical Journal:*

GENTLEMEN.—Dr. F. Peterson, House Physician, has at my request kindly furnished me from the records, a copy of the following remarkable case, which was under my charge last year at the Buffalo General Hospital. It deserves prominent notice, since recovery from the severe and complex injury, has no parallel, I believe, in the records of surgery. Yours truly, CHAS. C. F. GAY.

JOHN DEGENFELDER, entered Buffalo General Hospital, March 14, 1879; single; aged 18; was caught in belting and whirled several times around a shaft; injuries were as follows:

Fractured radius and ulna of right fore-arm at junction lower and middle thirds; fractured radius and ulna of right forearm at junction middle and upper thirds; fractured humerus of right arm at middle third; fractured clavicle of right side, near sternum; fractured femur of left side, comminuted.

Flesh wound exposing external hamstrings five inches in diameter. Wooden splints were applied to fore-arm after reducing swelling with hot fomentations. Swinburne's extension used on arm. These bones united in usual time; 2 inch shortening of humerus, and 1 inch of fore-arm; form of arm good. Compress and straps were put over clavicle; eight pounds extension applied to thigh; highest temp. 105.

April 21. Thigh swollen and fluctuating under pressure. Bistoury plunged in on outer surface, lower third, and one quart of pus was removed. Before next day two quarts more were removed. Whiskey, quinine and iron are his medications; Diet, milk, eggs, beef, beef-tea.

June 25. Still discharging several ounces daily of pus; femur seems to have united; pressure, by strapping, bandaging, wooden or tin splints tried; at one time seemed to have had pyæmia, pus-depot having formed in right arm, but ill omens soon passed away.

July 21. Put on plaster of Paris bandage.

Sept. 6. Able to drag himself over the building. Bones all united, but thigh crooked.

Sept. 26. Uses crutches and walks everywhere; able to go down town; health good; discharge from thigh small, thin and watery.

Feb. 12, 1880. Is now at work.

